# The “Naturalistic Free Recall” dataset: four stories, hundreds of participants, and high-fidelity transcriptions

**DOI:** 10.1038/s41597-024-04082-6

**Published:** 2024-12-03

**Authors:** Omri Raccah, Phoebe Chen, Todd M. Gureckis, David Poeppel, Vy A. Vo

**Affiliations:** 1 Department of Psychology, Yale University, New Haven, CT USA; 2 Department of Psychology, New York University, New York, NY USA; 3 Center for Language, Music, and Emotion, NYU & Max Planck Institute, Frankfurt, Germany; 4 Ernst Strüngmann Institute (ESI) for Neuroscience, Frankfurt, Germany; 5 Intel Labs, Intel Corporation, Hillsboro, OR USA

**Keywords:** Human behaviour, Perception

## Abstract

The “Naturalistic Free Recall” dataset provides transcribed verbal recollections of four spoken narratives collected from 229 participants. Each participant listened to two stories, varying in duration from approximately 8 to 13 minutes, recorded by different speakers. Subsequently, participants were tasked with verbally recalling the narrative content in as much detail as possible and in the correct order. The dataset includes high-fidelity, time-stamped text transcripts of both the original narratives and participants’ recollections. To validate the dataset, we apply a previously published automated method to score memory performance for narrative content. Using this approach, we extend effects traditionally observed in classic list-learning paradigms. The analysis of narrative contents and its verbal recollection presents unique challenges compared to controlled list-learning experiments. To facilitate the use of these rich data by the community, we offer an overview of recent computational methods that can be used to annotate and evaluate key properties of narratives and their recollections. Using advancements in machine learning and natural language processing, these methods can help the community understand the role of event structure, discourse properties, prediction error, high-level semantic features (e.g., idioms, humor), and more. All experimental materials, code, and data are publicly available to facilitate new advances in understanding human memory.

## Background & Summary

Since its inception, the empirical study of human memory has relied on controlled trial-based presentation of item lists, followed by an assessment of memory performance^1–3^. Despite the empirical utility and significance of these list-learning paradigms, researchers have increasingly argued for the use of naturalistic paradigms as a *complementary* source of evidence to ensure the broader applicability of findings generated under controlled conditions. This area of memory research primarily uses audiovisual movies and spoken narratives to uncover the impact of ecologically relevant variables and their interaction on manually annotated or automatically scored measures of memory performance^4–16^. This emerging literature has also resulted in a range of computational tools for annotating narratives and evaluating participant recollections^12,17–19^. Here, we contribute a naturalistic dataset to advance the study of human memory under ecologically valid conditions. Our work aims to expand the scope of questions that researchers can answer about human memory by (1) presenting the “Naturalistic Free Recall” dataset (NFRD), the most comprehensive dataset on human memory for narratives; (2) validating the use of this dataset for research on human memory by isolating classic signatures of human memory phenomena; and (3) providing researchers with a preview of methods and questions that can be answered with this type of data.

The NFRD contains data collected online from hundreds of participants who listened to a spoken narrative and immediately recalled it in as much detail as possible. Each participant was presented two of the four narrative stimuli included in the NFRD, and performed a spoken recall for at least 4 minutes following each story. This is intended to mimic real-world conditions for retelling narrative content. The dataset includes (1) text transcripts of the four distinct narrative stimuli, (2) professionally reviewed, high-fidelity transcripts of each verbal recall, and (3) time-stamps for each spoken word in the recall. While numerous studies have examined memory and perception for naturalistic content, verbal recollection - specifically in the form of storytelling - remains an understudied natural *behavior*. The retelling of narratives is a core part of our everyday lives, representing a fundamental human capability^20^. The NFRD provides a framework for studying which elements of a narratives are retained or forgotten by a listener, as well as how the encoded information is recalled verbally. We included four different narrative stimuli of a moderate length (average 11.58 minutes) to provide variability in a variety of stimulus features that may affect memory recall, such as semantic content, narrative structure, and emotional affect. This enables researchers to test the generalizability of empirical findings across multiple narrative contexts. Prior memory research has typically relied on data from approximately 20-30 participants recalling a single narrative or movie clip. In contrast, our dataset provides a substantially larger sample size, allowing researchers to investigate memory phenomena that may occur less frequently or reliably under naturalistic conditions (e.g., false memory, metacognitive abilities, etc.) The concern regarding low power for rare variables in naturalistic materials has been previously articulated by Hamilton and Huth^21^. Finally, the large sample size of our dataset allows for an analysis of individual differences in memory for narrative contents (e.g.,^22,23^). For example, this can be achieved by examining the relationship between recall features and factors such as age, particularly for the *oregontrail* and *baseball* narratives in the NFRD, which exhibit a wide distribution. Furthermore, we administered an open-ended questionnaire on mnemonic strategies that can be leveraged to investigate individual differences in learning outcomes^24,25^.

To validate the use of the NFRD for human memory research, we replicate well-characterized memory phenomena that have been reported in highly-controlled experimental paradigms. These classic list-learning paradigms have demonstrated serial position effects on memory^26–29^ and temporal contiguity effects^3,30–34^. In line with this extensive literature, we demonstrate that these signatures of memory recall are also present in the NFRD. This validation analysis relies heavily on previously developed computational approaches to study memory recall for narrative stimuli^12^.

Naturalistic studies of human memory provide us with the opportunity to answer novel questions that cannot be addressed with paradigms such as list learning. However, generalizing approaches from controlled experiments to naturalistic narratives introduces substantial conceptual and technical challenges. In traditional list-learning experiments, discrete items (e.g., words or images) have a clear match to particular items during recall. In contrast, real-world narratives lack such clarity in the elements that are remembered, and the correspondence between these elements and their recall is dependent on their conveyed meaning. For this reason, one primary obstacles with this work is the development of analysis tools to annotate the narrative stimuli and the spoken recall data. We conclude our paper with a concise review and partial application of the latest computational tools and methods that may be applied to analyze the NFRD. This includes automatic event boundary detection, a variety of semantic annotation tools, and automated approaches for scoring memory performance and features of memory recall. Future work may even use the NFRD to develop and validate novel computational tools for research on human memory.

## Methods

### Participants

The NFRD was collected from native English speakers who participated in the online study (N = 229; 145 female; *mean*_*age*_ = 25.03, *SD*_*age*_ = 11.15). The experiment was conducted across two distinct online platforms: the SONA Systems platform at New York University (N=167, *mean*_*age*_ = 19.67, *SD*_*age*_ = 1.33) and Prolific (www.prolific.com; N=62, *mean*_*age*_ = 39.77, *SD*_*age*_ = 12.90). Participants recruited via SONA were NYU undergraduates who received course credit for their involvement, whereas those recruited through Prolific represented a broader demographic and received compensation at a rate of $10 an hour. The study was approved by the local institutional review board (NYU’s Committee on Activities Involving Human Subjects; IRB-FY2016-1357). Before starting the online experiment, the consent form was presented on the screen and participants indicated using their keyboard that they agree to participate in the experiment. The consent form also included the following statement regarding data sharing: “Information without identifying details may be used in future research or shared with other researchers without additional consent.”

The data were derived from an originally collected participant pool of 291 participants through applying exclusion criteria to ensure data quality and gender balance. Specifically, 7 participants were excluded due to self-reported low engagement ratings (lower than or equal to 2 out of a 5-point Likert scale), 4 participants were excluded for missing age data, and 1 participant was excluded for missing questionnaire data. Additionally, data from 50 participants were not included in the current dataset due to budget constraints related to audio transcription. When excluding based on budget constraints, we employed a sample stratification strategy in which participants from non-male gender groups (female or other) were randomly selected, given the smaller number of male participants initially collected. We anonymized participant information by replacing names with numerical IDs. The only demographic information we have released is age and gender. Furthermore, we have not shared the audio recordings themselves to avoid re-identification risks and instead share recall transcripts with word-level timestamps (see following sections).

### Stimuli

The stimuli comprised four distinct spoken narratives in English. Three of the narratives were sourced from The Moth Radio Hour podcast series, specifically *Pieman, Eyespy*, and *Oregontrail*. The fourth narrative was the first chapter from the audiobook “Baseball Joe in the Big League” by Lester Chadwick, henceforth referred to as *Baseball*. This audiobook chapter was obtained from *LibriVox* (www.librivox.org). Each narrative was accompanied by a corresponding text transcript. The text for *Pieman* was gathered from a previously published neuroimaging dataset^4^, while the text for *Baseball* was publicly available on *Project Gutenberg* (www.gutenberg.org). For the remaining narratives, we followed the procedures outlined in the Speech-to-text Transcriptions section.

On average, each audio clip lasted 11 minutes and 35 seconds, inclusive of the 15 seconds of music preceding ”Pieman” and the 15 seconds of silence following it. All other audio clips began and ended promptly. The narratives contained an average of 1936 words each. A summary of the stimuli and participant demographics is presented in Table 1.

**Table 1:** Overview of the narrative stimuli and participant demographics.

	Stimulus source	Speaker gender	Length in words	Length in audio (seconds)	Participant N	Participant age range	Participant gender
*pieman*	The Moth Radio Hour	Male	948	489	116	17-29	88 female
*eyespy*	The Moth Radio Hour	Female	2318	779	116	17-29	88 female
*oregontrail*	The Moth Radio Hour	Female	2389	743	113	18-75	57 female
*baseball*	LibriVox Audio	Male	2088	768	113	18-75	57 female

### Task Procedure

The experiment was administered online and programmed using the PsychoPy library^35^. Participants were instructed to use headphones or earphones throughout the experiment. After the initial audio sound check, they listened to the verbal instructions, after which the same instructions were displayed as text on the screen. Participants were instructed to “recollect the story in as much detail as possible by speaking aloud.” While participants were encouraged to recall the story in the correct order, they were asked to return to any earlier points they might have missed. Participants’ responses for both the experiment and the questionnaire were collected using either their native computer keyboard or an external keyboard.

**Fig. 1:**
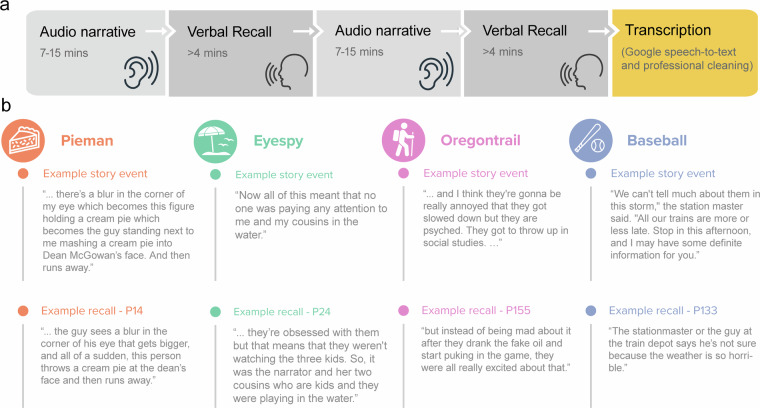
Experimental procedures and examples of narrative recalls. (**a**) Participants were instructed to listen to and subsequently recall two stories in as much detail as possible and in the correct order. Verbal recollections were automatically transcribed using Google Speech-to-Text and subsequently underwent manual cleaning by a professional transcription service. (**b**) Example events from each of the four narratives, along with their corresponding recollections by selected participants (participant IDs indicated in the titles).

Each participant was exposed to 2 out of 4 spoken narratives (Fig. 1). While the presentation order was randomized, the same two stories were consistently paired together (*Pieman* and *Eyespy*; *Oregontrail* and *Baseball*). After listening to each audio narrative, participants were immediately prompted to verbally recall the narrative for a minimum of 4 minutes. A green circle was displayed on the screen for the first four minutes of the recording, after which a yellow circle was displayed. Participants were instructed to recall the entire story but could only proceed after the circle had turned yellow. The JavaScript code used to present the online experiment, including the full instruction text and post-task questionnaire, is shared alongside our data repository^36^.

We encountered occasional data loss due to technical difficulties with the platform or the participant’s hardware setup, such as empty audio recordings, resulting in a loss of recall data for approximately 9.4% of the presented stories.

### Post-task questionnaire

After participants completed the experiment, a questionnaire was administered to assess participants’ understanding of the task and mnemonic strategies. The first three questions serve to assess subjects’ understanding and subjective difficulty of the task: “I understood the task instructions”, “I was engaged in the experiment” and “I found the experiment difficult”. Participants were instructed to provide responses to statements on a Likert scale, which included five balanced responses (strongly disagree, disagree, neither agree nor disagree, agree, or strongly agree). Next, participants were asked to provide open responses stating their strategy for memorizing the stories, subjectively memorable moments, and activities during the task. The open-ended questions were: (a) Did you use a particular strategy to memorize the story? (b) Were there any moments in either story that you found particularly memorable? If so, please describe them below. (c) Is there anything you think we should know about your experience taking our experiment? (d) What were you doing while listening to the stories? These questionnaire responses are included in the dataset.

### Speech-to-text transcriptions

We first utilized the Speech-to-Text API offered by Google Cloud (cloud.google.com/speech-to-text) to process all audio recordings. This generated a preliminary transcription of the audio recording, including some punctuation. Next, both the original audio recording and the preliminary transcription were submitted to a commercial transcript correction service (www.transcriptionwing.com). This process ensured that each transcript underwent thorough human review and manual correction. Human reviewers were provided with specific instructions to exclude filler words (e.g., “um”, “oh”) and eliminate word repetitions (e.g., “and and”). Additionally, they received a list of proper nouns featured in each story to maintain consistent spelling throughout the transcript. All numerical values were transcribed into words. Although reviewers were informed that letter case and punctuation were not critical, transcripts were typically returned with proper case and punctuation.

### Text speech alignment

We used the Penn Phonetics Lab Forced Aligner toolkit^37^ (p2fa) to align the audio files to words in the transcript. The audio files were first resampled to the optimal sample rate for the algorithm (11,025 Hz) with SoX v14.4.2 (using the *rate* effect instead of the deprecated *polyphase*). The alignment produces TextGrid^38^ files that denote the time associated with the start and end of each word in the transcript, as well as common noises during natural speech (breaths, coughs, laughter, short pauses, etc.) Documentation regarding the TextGrid format, along with Python code that can be used to extract data from these files is available on the OSF repository^36^.

### Automated scoring approach

To validate the use of this data to score memory performance for each narrative, we applied a recently published automated method by Heusser and colleagues^12^. This method uses a topic model to extract latent themes (or topics) from the narrative transcript as well as participants’ recollections. These themes are then used to partition these transcripts into discrete events and to assess memory performance. The following sections summarize this approach with minor adjustments to previously published methods.

#### Latent topic models for narrative segmentation

Following procedures from Heusser *et al*.^12^, we extracted topic model vectors from both the narrative transcripts and recollections by applying Latent Dirichlet Allocation (LDA)^39^ on sliding windows of the text. We then segmented the sequence of topic vectors into discrete events with a Hidden Markov Model (HMM). We refer to this as the LDA-HMM method. Our sole modification to the Heusser *et al*.^12^ procedure involved adjusting the LDA hyperparameters via grid search to better suit our data.

Specifically, the grid search aimed to find the optimal combination of LDA hyperparameters that would result in high alignment between the automated narrative segmentation and human ground truth segmentations on a previously published, independent dataset collected on the *Pieman* story (N=205)^40^. We computed ground truth on the event segmentations by quantifying participant agreement across the 205 annotators, who pressed a button each time they encountered an event boundary while listening to the story. For each participant, we first downsampled the original 1000 Hz binary response vector provided by Michelmann *et al*.^5^ to a 0.2 Hz binary response vector. Any button presses within each 5 second window was coded as a “1” in the downsampled response vector. Our assumption is that events in the story would be at least 5 seconds (~10 spoken words) apart from one another. To determine statistical significance, we used a block permutation test on the downsampled response vectors, similar to Silva *et al*.^41^. We shuffled all participants’ response vectors across time, averaged them for 1000 iterations to generate a null distribution, and compared the initial averaged response to its 95th percentile (*α* = 0.05, 1-tailed test). Finally, we aligned each significant time window to a word in the narrative by identifying the closest word to the highest participant agreement peak in the original 1000 Hz response vectors. We then manually adjusted these boundaries to the nearest sentence boundary, and any overlapping events after this adjustment were merged, following Michelmann *et al*.^17^.

Then, we returned to optimize the LDA-HMM alignment with ground truth by searching across 3 LDA hyperparameters: sliding window size (25 to 100 words), step size (1 to 50 words), and topic vector dimension (10 to 100). The optimal combination was a 55-word window, 21-word step size, and 40-dimension topic vector, which achieved a F1 score of 0.72 in matching the LDA-HMM segmentations to the ground truth segmentations. Importantly, we apply the hyperparameters derived from the Pieman narrative to all stories, under the assumption that they will capture event segmentation patterns across a wider spectrum of narratives.

#### Probability of recall

We next sought to compute the overall probability of recollection for events in each narrative. For each recall event, we found the best corresponding story event by identifying the highest correlation across recall-story topic vectors. This approach allows for some story events to not have a corresponding recollection. As such, our analysis assumes that not all events in the story are recalled by a participant. We consider story events with no matching event in the recall as forgotten by the participant. This is in contrast to Heusser *et al*.^12^ which matches narrative events to participant recollections, ensuring that each event has one or more corresponding recall. Conceptually, we believe it is reasonable to assume that some events may not be recalled at the participant-level.

We then computed probability of recall, i.e., percentage of participants who recalled each story event, instead of the average precision (i.e., correlation coefficient) values reported in^12^. To do so, we constructed a matrix in which rows represented participants and columns represented story events, with values set at 0 for events not recalled and 1 for those recalled. We then averaged across participants to construct a continuous measure of recall probability. We then computed 95% confidence intervals across participants by bootstrapping each event, resampling participants with replacement 10,000 times.

**Fig. 2:**
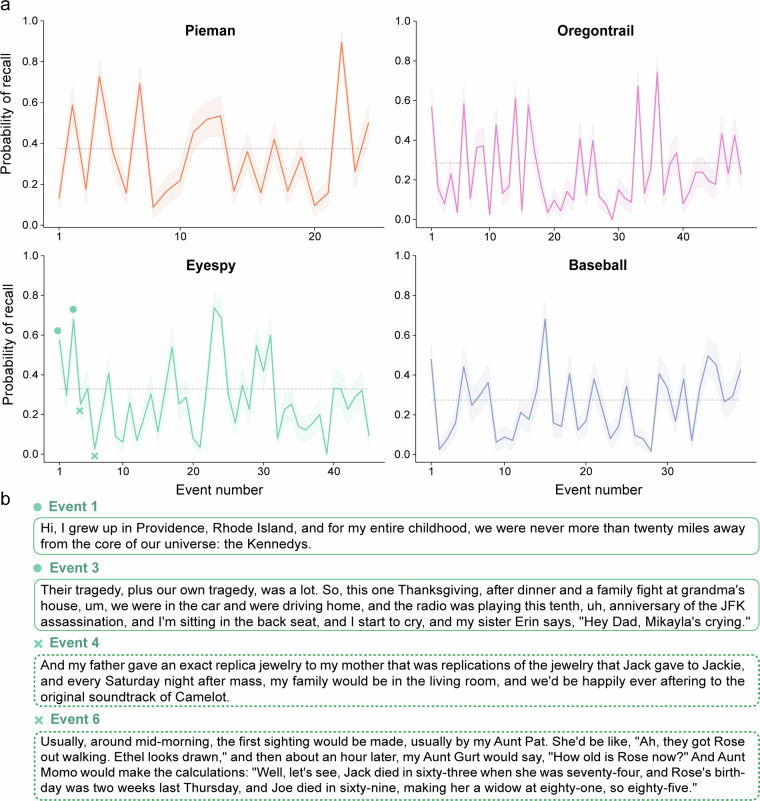
Quantifying narrative event recall. (**a**) Probability of recall for all the events in four stories, averaged across participants. The shaded error bars represent bootstrap 95% confidence intervals. We computed a permutation baseline that quantifies the mean event recall for each story. Events above the 97.5 percentile (dotted gray line) are recalled significantly more often than the typical event in that story (percent of significantly recalled events: Pieman 37.5%; Eyespy 31.1%; Oregontrail 30.6%; Baseball 43.6%). (**b**) Example story events from Eyespy that were recalled more than average (the first two marked by dots) or less than average (the latter two marked by crosses).

To test whether an event was significantly recalled across participants, we used a permutation test. To construct a null distribution, we permute the order of the events within each participant and re-compute the average probability of recall for each event. We apply 10,000 repetitions to ensure a reliable estimate of the null distribution. Importantly, this approach assumes that the base rate of recalling each event is independent of its position in the story. For a 2-tailed test at *α* = 0.05, an event is significantly recalled if the average recall probability exceeds the 97.5th percentile of the null distribution (Fig. 2).

#### Extending list learning effects

**Fig. 3:**
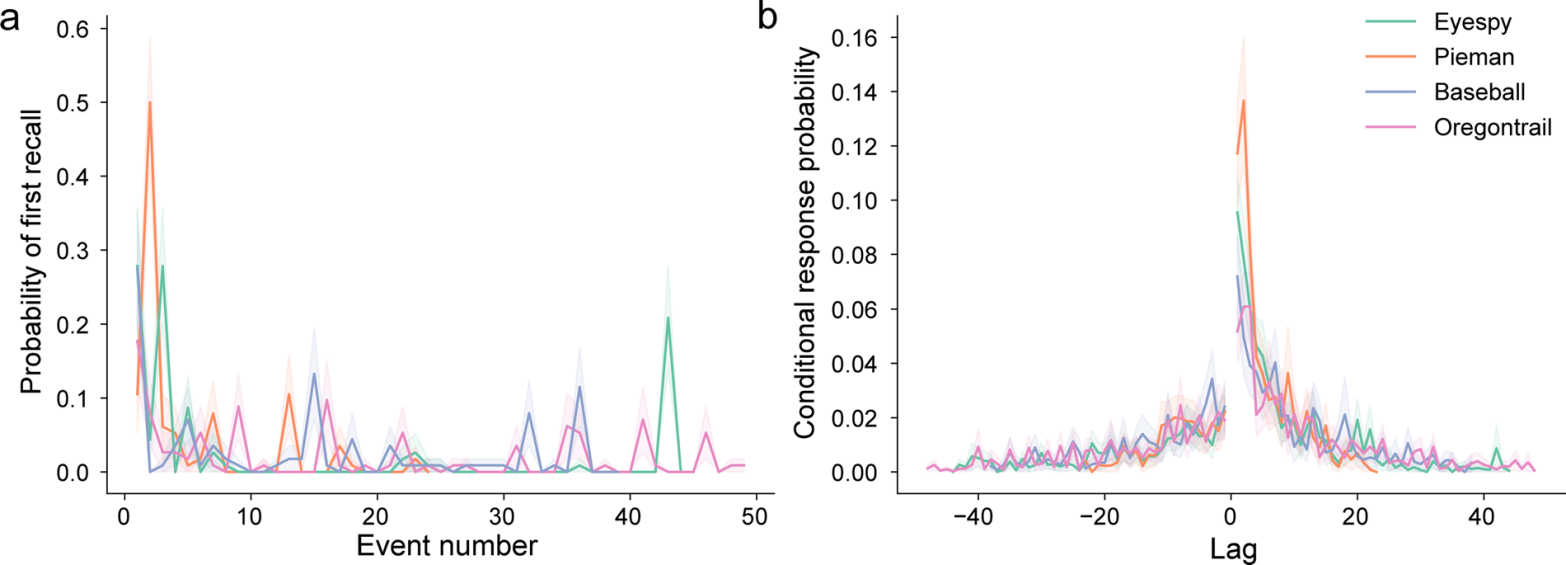
Extension of classic list-learning effects under naturalistic conditions. **a**) The probability of first recall as a function of serial position of an event in each narrative. **b**) The conditional probability of recalling each event following all other events, plotted for each spoken story. The shaded error bars represent bootstrapped 95% confidence intervals.

As part of the technical validation, we sought to extend key effects from the list-learning literature^31^. Specifically, we analyze the probability of any given event to be recalled first (probability of first recall; PFR) and whether events with close temporal proximity are recalled successively (lag-conditional response probability; lag-CRP). For both measures, we used the probability of recall values for each narrative event. To generate the PFR function, we computed the probability of events getting recalled first depending on their serial position. To generate the lag-CRP function, for every pair of consecutively recalled events, we computed the distance between their corresponding story events and counted the number of pairs in every possible lag. The count is then normalized within participant, and then averaged across participants to generate the conditional response probability. For the first event in PFR and one-lag probability in CRP, we computed the significance values from permutation distributions that was created by permuting the recall order within participants (10,000 repetitions; Fig. 3).

## Data Records

The data is publicly available on the Open Science Framework (OSF) data repository (https://osf.io/h2pkv/) under a Creative Commons License (CC0 1.0 Universal)^36^. The dataset is organized in two primary directories. The first is experiment_materials, which contains information pertinent to the stimuli and experimental design. Specifically, it contains a text file with the experiment instructions, a psychopy folder housing the task code deployed on both Prolific and SONA systems, and a stimuli folder containing audio files and transcripts of the four stories. The second directory, titled data, contains cleaned recall transcripts recall_transcripts) and time-aligned transcripts recall_aligned) for each participant, sorted according to the respective stories. Lastly, the “survey” subfolder contains demographic details and questionnaire responses for all participants.

## Technical Validation

We ensured the quality of the collected data by (1) applying exclusion criteria based on self-reported engagement and (2) using a commercial service to guarantee that a human reviewed each audio recording and corresponding text transcript to correct errors arising from automatic transcription. We further validated our dataset by applying previously developed methods to quantify event recall in audiovisual narratives^12^ to our dataset. We then use these metrics to extend classic effects from list learning literature in each narrative in the dataset.

### Quantifying average event recall

Using the LDA-HMM procedure reported in the Methods section, we computed the probability of recall across participants for each event in the four narratives. This analysis yielded recall curves for each narrative, providing a continuous measure of memorability for individual events. The y-axis in Fig. 2 denotes the percentage of participants that recalled a given event. These plots demonstrate substantial variability in event memorability, with some events being reliably recalled across participants, while others were inconsistently or infrequently recalled.

Future work can utilize these curves to explore relationships between event recall and various semantic features or subjective reports associated with specific narrative segments. Such inquiries are consistent with established approaches within the memory literature. Due to the large number of participants in the dataset, future work would be able to estimate these effects with relatively narrow confidence intervals.

### Extending list learning effects

Using the probability of recall curves, we next sought to extend classical list learning effects to our narrative stimuli, as demonstrated in^12^. Specifically, we investigated the probability of first recall^26–29^ and temporal contiguity effects^3,30–34^, which both characterize the order in which participants recall items from a list. In agreement with the literature, we found that the initial event in each narrative showed a significantly higher probability of being recalled first by participants (*Pieman*: $\bar{X}$ = 0.11, *Eyespy*: $\bar{X}$ = 0.28, *Oregontrail*: $\bar{X}$ = 0.18, *Baseball*: $\bar{X}$ = 0.27; *p* < 0.001 for all four stories; Fig. 3a).

Previous reports have shown that items from memory are typically recalled in the order that they were initially presented. They often characterize this effect by computing a conditional response probability curve across temporal lags. For some item *i*_0_, it shows the probability that nearby items will be recalled in order, e.g., item *i*_1_ will be recalled at the first lag, followed by item *i*_2_ at the second lag. Our results show a temporal contiguity effect across all narratives. There is a significantly higher probability at lag one (*Pieman*: $\bar{X}$ = 0.12, *Eyespy*: $\bar{X}$ = 0.10, *Oregontrail*: $\bar{X}$ = 0.05, *Baseball*: $\bar{X}$ = 0.07; *p* < 0.001 for all four stories; Fig. 3b), which diminishes as the lag positively increases. This has previously been shown for a audiovisual narrative^12^, but we show here that it also extends to purely audio narratives. Interestingly, we find some variability across narratives, suggesting that there may be factors in the content or structure of these narratives that impact the order of recall.

The successful extension of these two classically documented memory effects gives us confidence in the quality of this dataset.

## Usage Notes

One of the key challenges with naturalistic experiments is the difficulty in analyzing and interpreting the results. To enable the community to make effective use of this dataset, we highlight computational methods used to represent complex properties of narratives, with a focus on their use in studies of human memory. We encourage the research community to continue the use and development of these tools to answer new research questions using narrative stimuli.

### Automated event segmentation

The structure of events in a narrative is considered crucial for our episodic memory^42^. Recent studies have explored various methods to automatically segment events from narrative text. While most of the work relies on HMMs^12^, careful LLM prompting can generate event boundaries that are more representative of the average rater than individual human participants^17^. Once segmented, narratives can be analyzed to uncover properties and relationships among events that may influence memory retention. For instance,^43^ found that LLMs can accurately score the plausibility of different agent-patient interactions in events.

### Semantic and discourse properties

Annotating the semantic and discourse properties in narratives requires methods that can integrate over long temporal windows (i.e., spanning multiple words, sentences, or paragraphs). Recent approaches have drawn upon methods from linguistics and natural language processing (NLP) to represent the semantic content of narratives. These include latent topic models^44^, sentence embedding methods^13,19^, and more recently, autoregressive language models (LMs). As machine learning methods continue their rapid pace of development, researchers can anticipate a variety of new methods for annotating complex semantic properties. For instance, recent work investigated the viability of large language models (LLMs) to code different categories of interest in sociolinguistics, such as figurative expressions (e.g. sarcasm, idioms, etc.) and humor^45^. While it was reported that pre-trained LLMs showed promising results, further development is needed before such models can be used in a completely unsupervised manner.

**Fig. 4:**
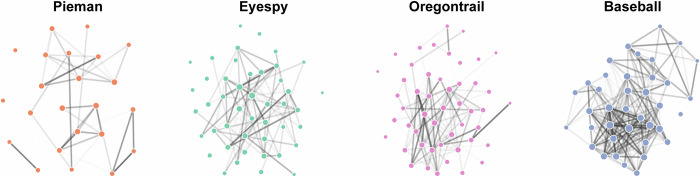
Semantic narrative networks. To construct these semantic networks, embedding vectors were generated for text in each narrative event using Google’s Universal Sentence Encoder^53^. In generating these network diagrams, we threshold the cosine similarity at 0.35 between events to generate the edge weights. The thickness of the lines are proportional to these weights. The size of the nodes (representing events) reflects the semantic centrality across relationships, independent of a threshold. These networks were applied to the NFRD following the methods in Lee and Chen^46^.

In a recent study by Lee and Chen^46^, sentence embedding techniques were used to construct undirected graphs representing the relationships between narrative events based on their semantic similarity. We apply these methods to the NFRD as displayed in Fig. 4. Following the procedures from Lee and Chen^46^, we find a significantly positive effect of semantic centrality - indicating the semantic similarity of an event to all others within the narrative - on the likelihood of recall for that event (*p* < 0.001 in a linear mixed-effects model); notably, the effect size (*β* = 0.27) mirrors that reported in Lee and Chen^46^. This replication of these high-level semantic effects using a fully automated scoring approach^12^ underscores the robustness of the dataset utilized in our study. We provide all code utilized in the application of these methodologies to the current dataset to facilitate future research.

Autoregressive LMs, leveraging contextual information to generate predicted probability distributions for subsequent tokens or words, have also been utilized by researchers to compute a continuous measure of prediction error or surprise. This approach incorporates more semantic information than other measures of surprise^47^. Prediction error, or surprise, has been shown to influence memory encoding and event segmentation^6,48,49^. These developments in NLP have allowed researchers to investigate how these model-based definitions of prediction error impact memory behavior, rather than relying on experimental manipulations of prediction error^10,50^.

Recent work has developed LLM-based methods to score other properties of the memory recall. For example, a recent study fine-tuned an LLM to distinguish recall details pertaining to the central event versus those that are unrelated^18^, replacing a more laborious interview method to score autobiographical recall^51^. This method has been used to understand how surprise and emotion impact autobiographical memory^52^.

## Data Availability

The code is available on https://github.com/phoebsc/Narrative-Memory-Dataset. The code is in Python, using standard packages such as NumPy and SciPy, and topic modeling functions from Heusser *et al*.^12^.
